# One Identity or More for Telomeres?

**DOI:** 10.3389/fonc.2013.00048

**Published:** 2013-03-15

**Authors:** Marie-Josèphe Giraud-Panis, Sabrina Pisano, Delphine Benarroch-Popivker, Bei Pei, Marie-Hélène Le Du, Eric Gilson

**Affiliations:** ^1^Faculté de Médecine de Nice, Université de Nice-Sophia Antipolis, Institute for Research on Cancer and Aging Nice, UMR 7284 CNRS, U1081 INSERMNice, France; ^2^CEA/DSV/IBiTec-S/SB2SM, Laboratoire de Biologie Structurale et Radiobiologie, CNRS UMR 8221, University Paris-SudGif-sur-Yvette, France; ^3^Department of Medical Genetics, Archet 2 Hospital, Centre Hospalier Universitaire de NiceNice, France

**Keywords:** telomeres, capping complexes, telomeric chromatin organization, DNA topology

## Abstract

A major issue in telomere research is to understand how the integrity of chromosome ends is controlled. The fact that different types of nucleoprotein complexes have been described at the telomeres of different organisms raises the question of whether they have in common a structural identity that explains their role in chromosome protection. We will review here how telomeric nucleoprotein complexes are structured, comparing different organisms and trying to link these structures to telomere biology. It emerges that telomeres are formed by a complex and specific network of interactions between DNA, RNA, and proteins. The fact that these interactions and associated activities are reinforcing each other might help to guarantee the robustness of telomeric functions across the cell cycle and in the event of cellular perturbations. We will also discuss the recent notion that telomeres have evolved specific systems to overcome the DNA topological stress generated during their replication and transcription. This will lead to revisit the way we envisage the functioning of telomeric complexes since the regulation of topology is central to DNA stability, replication, recombination, and transcription as well as to chromosome higher-order organization.

## Forewords

Through 70 years of studies of the terminal part of linear chromosomes, the telomeres, much has been learned about the specificity of these critical genetic elements. Telomeres are necessary to protect chromosome ends from unwanted activation of the DNA damage checkpoint, inappropriate repair, and nucleolytic degradations. Surprisingly, the molecular solutions selected through evolution for this protection show a remarkable degree of diversity. In this review, we attempted to compare the different levels of telomere organization found in a variety of organisms in the hope of revealing universal themes that characterize telomeres structure and function.

## Telomeric DNA: Repetitive in Nature, Diverse in Organization, and Sequence

Telomeric DNA is constituted of repetitive sequences that can adopt different conformations and exhibit variable length across species. In vertebrates a six nucleotide sequence T_2_AG_3_ is repeated over a few hundred bp to more than 50 kb in some mammals. If size varies greatly, the G-rich nature of the telomeric motif is usually much conserved.

Telomeres shorten at each cell division because of the inability of DNA polymerases to replicate linear DNA to completion. Ultimately, critically short telomeres become non-functional which leads to a telomere-dependent cell cycle arrest termed replicative senescence. In several cell types (in humans: stem cells, cancer cells, or the germline) or organisms, this is counteracted, to different degrees, by elongation which is mostly performed by a telomere-specific reverse transcriptase, the telomerase enzyme. When telomerase activity is low or absent, telomere length is maintained by a recombination-based system (Alternative Lengthening of Telomeres, ALT). Notable exceptions are dipterans where telomeres are elongated by insertion of retrotransposons to the chromosome ends. The better studied example is *Drosophila melanogaster* where terminal repeats consist of three telomere-specific non-long terminal repeat retrotransposons, Het-A, TART, and TAHRE, called HTT arrays (Mason and Biessmann, 1995; Mason et al., 2008). Variations also exist within dipterans. Indeed, in *Rhynchosciara americana* (a lower dipteran), a composite structure of a retrotransposon and an AT-rich repeat motif called RaTART has been reported (Madalena et al., 2010).

Telomeric DNA usually terminates with a single strand overhang. Mammals, *Saccharomyces cerevisiae*, and *Oxytricha* harbor a 3′ G-rich overhang (Makarov et al., 1997; Hemann and Greider, 1999; Jacob et al., 2003). The length of this single strand varies between organisms from 16 nt in ciliates to between 100 and 300 nt in mammals (Henderson and Blackburn, 1989; Wellinger et al., 1993a,b; Makarov et al., 1997; Jacob et al., 2003; Lee et al., 2008). In *S. cerevisiae*, a G-strand overhang can be detected during most of the cell cycle but longer G-tails were observed transiently in late S phase, when telomerase was shown to be active (Wellinger et al., 1993b; Marcand et al., 2000). Lagging strand synthesis leads to an overhang that is eventually elongated further by telomerase. Leading strand synthesis, however, generates a blunt end that has to be resected. In mouse cells, telomeric 3′ overhangs originate from the concerted post-replication action of two exonucleases, Exo I and Apollo under the surveillance of POT1b, the single strand DNA binding protein from the Shelterin complex, that inhibits any excessive resection and recruits the CST complex for a proper correction of the generated overhangs (Lam et al., 2010; Wu et al., 2012). The shelterin or shelterin-like and the CST or CST-like complexes are telomeric proteins complexes that are found in many species. In vertebrates, the shelterin complex is organized around six proteins, TRF1, TRF2, RAP1, TIN2, TPP1, and POT1 (in some species such as mice one can find two POT1 proteins). As its name suggests, this complex and several of its sub-complexes are involved in telomere protection against illegitimate repair, untimely degradation, or cell cycle checkpoint activation (more about the shelterin can be found in a later section of this review). Originally found in *S. cerevisiae*, the CST (Cdc13-Stn1-Ten1) complex binds to the single strand terminal tail regulating its formation and thus participates in telomere capping (Giraud-Panis et al., 2010b).

Alternative end-structures can also exist as described for *C. elegans* which telomeres contain both 3′ G-strand and 5′ C-strand overhangs (Raices et al., 2008). C-overhangs have also been detected in mammalian cells. They are more abundant in G1/S phase in arrested and in terminally differentiated cells (Oganesian and Karlseder, 2011). Finally, blunt-ended telomeres have been described in plants, illustrating further the diversity of DNA organization found at chromosome ends (Kazda et al., 2012).

Such a diversity of repetitive sequences and structures between organisms is also found for another basic element of chromosomes: the centromere. In this case, it was proposed that the main determinants of centromeric functions do not directly rely on their DNA sequence but on epigenetic mechanisms. Indeed, in most species centromeres are not genetically defined but are marked by a specialized histone 3 variant (CENP-A in mammals) which is the key determinant of kinetochore assembly (Allshire and Karpen, 2008).

Interestingly, the involvement of epigenetic determinants might also hold true for telomeres since the protection of chromosomal DNA ends in *Drosophila* can be achieved in a DNA sequence independent manner (Raffa et al., 2011).

## Folding of the Single-Stranded Telomeric DNA: Wanted or Unwanted Conformations?

**Figure 1:**
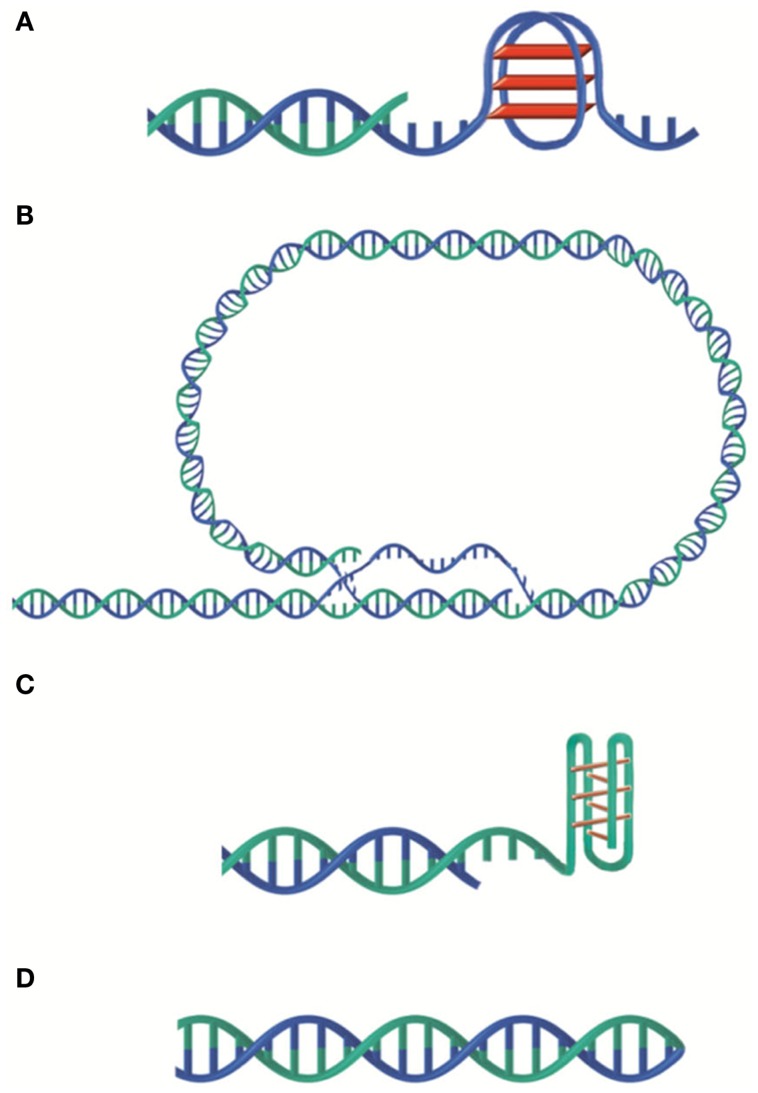
**Versatility of telomeric DNA structures**. **(A)** G-quadruplex. **(B)** t-loop. **(C)** i-motif. **(D)** G-C hairpin end. G-rich and C-rich strands are represented in blue and green respectively.

In addition to its role as telomerase substrate, the G-rich 3′ overhang found in many organisms can form G-quadruplex (G4) structures consisting in cyclic planar arrangements of four hydrogen-bonded guanines that stack on top of each other (Figure 1A). On telomeric DNA, this might happen within the G-overhang, on any single strand that may appear during replication or at the D-loop located at the foot of the t-loop (Figure 1B). Formation of transient G4 structures could hinder lagging strand synthesis giving a possible explanation for the requirement of the Werner helicase for efficient replication of human telomeres (Crabbe et al., 2004). A similar mechanism is thought to be possible during transcription (Duquette et al., 2004). G4 structures could also participate in the dynamics of telomeric DNA melting. One could imagine that transient G4 may help double strand opening perhaps explaining why T_2_AG_3_ sequences are prone to single strand invasion (Amiard et al., 2007).

It is also known that G-quadruplex inhibits telomerase activity and recently it has been found that a pentacyclic acridinium compound that stabilizes G4 structures, RHPS4, in synergy with camptothecin or a PARP-1 inhibitor leads to complete tumor regression in mice (Salvati et al., 2007, 2010; Leonetti et al., 2008). G4 may thus constitute promising targets for cancer therapy. Interestingly it has been found that in *Drosophila*, Het-A contains sequences also allowing G-quartets formation *in vitro* (Abad and Villasante, 1999). In addition, telomeric proteins such as POT1 from human telomeres and TEBP from *Tetrahymena* have been shown to be able to control the formation of G-quadruplex DNA structures *in vitro* and *in vivo* respectively (Paeschke et al., 2005; Zaug et al., 2005; Torigoe and Furukawa, 2007).

C-rich single strands can also form peculiar structures such as i-motif (Esmaili and Leroy, 2005) although, in that case, the acidic pH required for its formation may be rarely obtained in physiological conditions (Figure 1C). G and C-rich telomeric single strands have been shown to form G–G and C–C hairpin structures in *Oxytricha nova* and *Tetrahymena* sequences *in vitro* (Ahmed and Henderson, 1992; Laporte and Thomas, 1998; Krafft et al., 2002). More classical G-C hairpins (Figure 1D) have also been observed. Indeed, in *Borrelia* linear plasmids, the two telomeric strands are linked at the ends thus forming a hairpin loop (Hinnebusch and Barbour, 1991).

## The t-Loop Model or How to Translate a Linear Problem into a Circular One

The 3′ overhang can also allow the folding back of the telomeric DNA into a loop, called t-loop (Figure 1B). Jack Griffith and colleagues showed the presence of t-loops in telomeric DNA of several organisms, mammals (Griffith et al., 1999; Cesare and Griffith, 2004), trypanosome (Munoz-Jordan et al., 2001), fission yeast (Tomaska et al., 2004), garden pea (Cesare et al., 2003), bacteria with linear genome (Cesare et al., 2008), and yeast mitochondria (Tomaska et al., 2002). These loops were also visualized in telomeric chromatin extracted from chicken erythrocyte and mouse lymphocyte nuclei (Nikitina and Woodcock, 2004). This DNA structure was proposed to result from the invasion of the single strand overhang into the double strand in a *cis*-oriented reaction followed by migration leading to the formation of both a D-loop and a Holliday junction (Griffith et al., 1999; Amiard et al., 2007; Poulet et al., 2009). Of note, *in vitro* assays showed that both the G-rich and the C-rich strands can invade a double-stranded-DNA and are both able to form t-loops (Verdun and Karlseder, 2006; Raices et al., 2008). Also *in vitro*, TRF2 is necessary and sufficient to produce t-loops (Griffith et al., 1999; Stansel et al., 2001; Yoshimura et al., 2004) probably thanks to its capacity to stimulate single strand invasion and to protect and favor Holliday junctions (Amiard et al., 2007; Poulet et al., 2009). In cells, deletion of the N-terminal basic domain of TRF2 causes a significant decrease in telomere length and the formation of telomeric DNA circles that are thought to be produced by the processing of the t-loop (Wang et al., 2004; Vannier et al., 2012). The function(s) of t-loops are unknown. It is inferred from the capacity of t-loop to hide the terminal 3′ overhang and from the role of TRF2 in both end protection and t-loop formation that t-loop could protect chromosomes ends from being recognized as an accidental double strand break. In addition, t-loops might regulate telomerase access to DNA and initiate intratelomeric recombination events.

## Telomeric RNA: No Exception Yet

The first indication that telomeric repeats could be transcribed stems from studies in *Trypanosoma* (Rudenko and Van der Ploeg, 1989). This notion was then extended to mammalian telomeres where it was shown that telomeric C-strands were transcribed by RNA polymerase II into a non-coding telomeric repeat-containing RNA called TERRA (Azzalin et al., 2007; Schoeftner and Blasco, 2008; Porro et al., 2010). Since then, telomeric RNA have been described in many species such as birds (Solovei et al., 1994), budding yeast (Luke et al., 2008), *Arabidopsis* (Vrbsky et al., 2010), or fission yeast (Greenwood and Cooper, 2012) suggesting overall that transcription of telomeres is a conserved phenomenon through evolution.

Mammalian TERRA ranges from 100 bp to 9 kb and contains a 7-methylguanosine cap structure and a polyA tail on a fraction of the transcripts. Only the polyA^−^-RNA are associated to chromatin. Interestingly TERRA expression varies during the cell cycle, with a low level in late S phase and a high level in early G1-phase (Porro et al., 2010).

**Figure 2:**
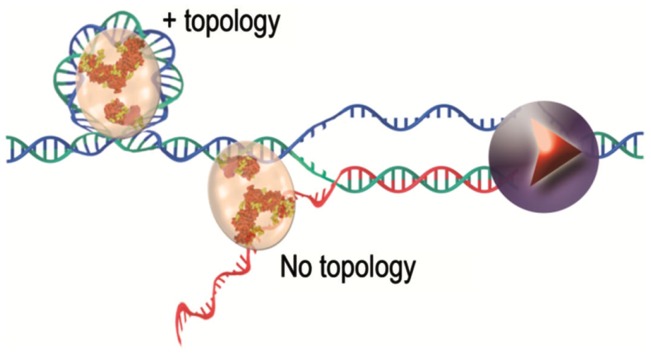
**TRF2 protein complexes with nucleic acids, a working model**. TRF2 binds to DNA and modifies its topology. This intrinsic property of TRF2 is inhibited by the binding of TERRA (in red in the figure) synthesized by RNA polymerase II (represented in violet).

Telomeric RNA function remains unclear but several ideas have been proposed:

i) TERRA could play a role in telomerase regulation. (UUAGGG)_3_ RNA oligonucleotides inhibit telomerase activity *in vitro* probably through interactions with the template part of telomerase RNA (Schoeftner and Blasco, 2008). In budding yeast, the formation of a DNA/RNA hybrid between TERRA and telomeres is also thought to inhibit telomerase action (Luke et al., 2008). However, this view was recently challenged by the observation that telomerase activity is not restrained at highly transcribed telomeres (Farnung et al., 2012).
ii) TERRA could be part of specific telomeric nucleoprotein structures (Deng et al., 2009). Indeed, the telomeric protein TRF2 binds TERRA via the G-quadruplex structures it can form (Biffi et al., 2012). Furthermore, small RNAs mimicking TERRA have been shown to bind to TRF2 and to inhibit TRF2 ability to modify DNA topology (Poulet et al., 2012) (Figure 2). Thus, G4 formation together with TRF2/TERRA interaction might constitute another way of regulating telomerase access to chromosome ends and/or of regulating telomeric factors during the different processes of transcription or replication.
iii) TERRA could contribute to telomere protection. RNAi-depletion of TERRA induces the recruitment of DNA damage response factors on telomeres suggesting a role of TERRA in telomere protection and stability (Deng et al., 2010).
iv) TERRA could play a role in heterochromatin formation. In favor of this hypothesis, TERRA accumulates in close vicinity of the inactive X chromosome in female mouse cell lines (Schoeftner and Blasco, 2008), it accumulates near both X and Y chromosomes in stem cells (Zhang et al., 2009) and its downregulation in human cell lines, leads to a loss of histone H3K9me3 (Deng et al., 2009). Recently, human TERRA has been shown to be able to regulate its own transcription (Arnoult et al., 2012).

In fission yeast, transcription of the G-strand into a C-rich telomeric RNA has been observed. This RNA, called ARIA, is more abundant than the G-rich RNA (Greenwood and Cooper, 2012). ARIA does not contain subtelomeric sequences and is not synthesized by RNA pol II. It should be generated via promoter sites composed of telomeric repeats (Bah et al., 2011; Greenwood and Cooper, 2012). A specific role of ARIA has not been described so far but we can hypothesize that it could act as a regulator of TERRA or of the 3′ overhang by hybridizing to these RNA/DNA single strands.

Non-telomeric RNAs are also produced from subtelomeres in budding and fission yeasts as well as in plants. This transcription is bidirectional, resulting in ARRET and α-ARRET molecules (Vrbsky et al., 2010; Bah et al., 2011; Greenwood and Cooper, 2012). In *Arabidopsis thaliana*, interestingly, TERRA and ARRET RNAs can hybridize and be processed by Dicer into short siRNAs (Vrbsky et al., 2010).

## Capping Proteins: Diversity and Plasticity are the Rules

**Figure 3:**
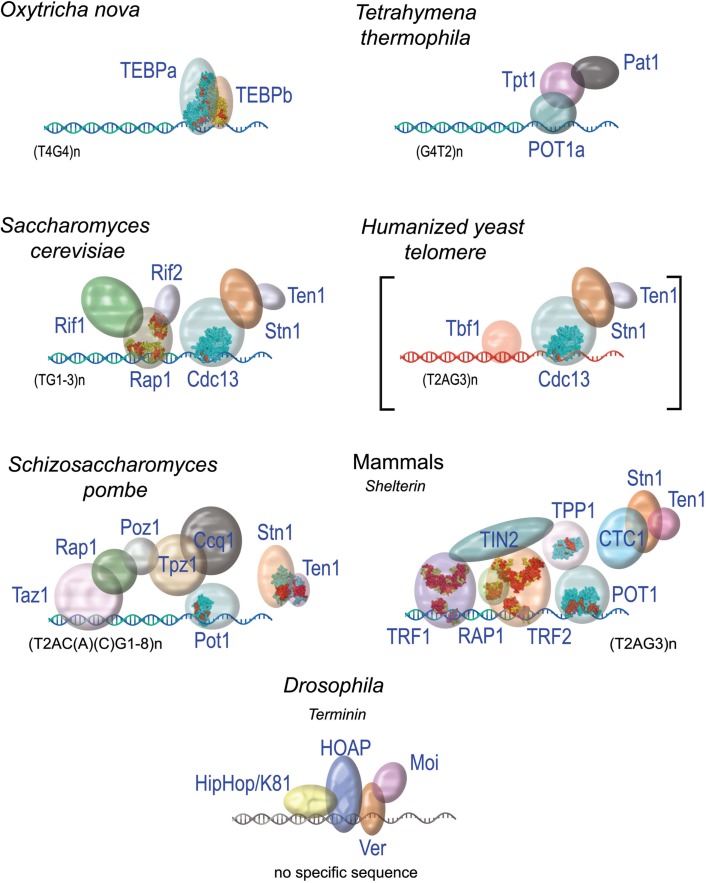
**The diversity of protein capping complexes in different species**. When solved, the 3D structures of proteins or domains are shown (for pdb entry numbers refer to Giraud-Panis et al., 2010a).

It is now clear that telomere functions are achieved, in large part, through the recruitment at chromosome ends of specific capping proteins (Sfeir and de Lange, 2012). Surprisingly enough for such an essential function, the organization of these capping proteins can greatly vary between organisms (Figure 3). A single protein heterodimer, TEBPα and β that form a tight ternary complex with the 3′ overhang or telomeric DNA was found in *O. nova* (Gottschling and Zakian, 1986; Price and Cech, 1987). The capping system of *S. cerevisiae*, which includes both a ss-DNA binding complex CST (for Cdc13-Stn1-Tel1, Giraud-Panis et al., 2010b) and a ds-DNA binding complex is centered on the protein Rap1. In fission yeast and in mammals, the capping protein complex (named Shelterin) bridges the ss- and the ds part of telomeric DNA. The recruitment of these protein complexes at chromosome ends is due to the specific recognition of telomeric DNA sequences by some of their subunits. In the human Shelterin complex, TIN2 bridges TRF1 and TRF2 (Ye et al., 2004), and forms a complex with TPP1 and POT1 in the order TIN2-TPP1-POT1 (O’Connor et al., 2006; Wang et al., 2007). Therefore, the interaction between the proteins TPP1 and TIN2 forms the keystone of the bridge between ss-DNA and ds-DNA. A similar organization is found in the terminal *Schizosaccharomyces pombe* complex that also contains six proteins (reviewed in Moser and Nakamura, 2009).

In the budding yeast *S. cerevisiae*, the CST complex located at the ss-DNA and the Rap1-Rif-Sir complex, on ds-DNA, seem to function independently. A direct interaction between Sir4 and Cdc13 has been observed *in vitro*, but this interaction does not seem to impact the function of each protein (Lewis et al., 2004). Functional regulatory interplay have been described through the ATM homolog Tel1, the MRX component Xrs2, or telomerase components, but no stable bridging interaction has been described.

It is worth noting that budding yeast telomere can be stably maintained in the absence of Rap1 binding in mutant cells expressing a sequence variant of the telomerase RNA template leading to the incorporation of T2AG3 repeats instead of the TG1-3 repeats at yeast telomeres (Alexander and Zakian, 2003; Brevet et al., 2003; Berthiau et al., 2006). This reveals an extraordinary plasticity for the mechanisms that protect chromosome ends. This could explain the diversity of capping proteins found in various organisms and suggests that common themes exist between seemingly different capping systems. This is particularly true considering the way these proteins interact with their corresponding DNA substrates.

**Figure 4:**
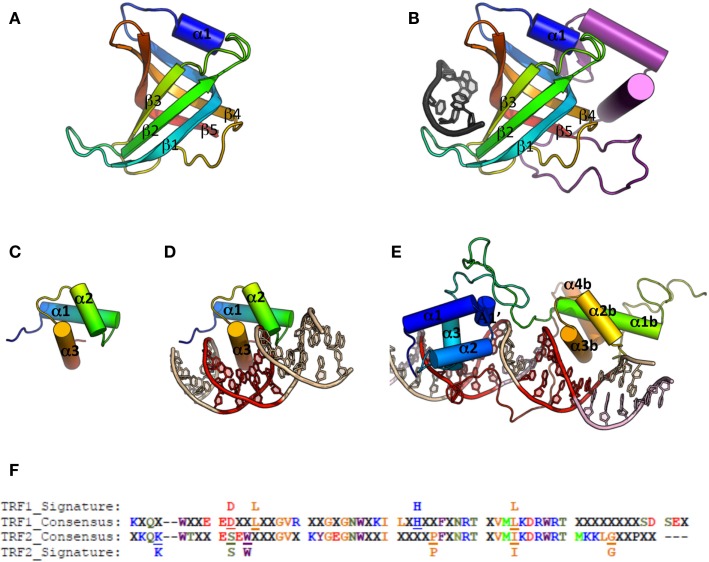
**Cartoon representations of ss-DNA and ds-DNA binding domains**. **(A)** Canonical OB-fold, with β-strand 1–5 and α-helix 1 (pdb entry 1QZG). **(B)** OB-fold from human Pot1 (1QZG) in complex with ss-DNA (dark gray), the additional secondary structures is shown in purple. **(C)** Canonical Myb-fold, with α-helix 1–3 (1W0U). **(D)** Myb domain from human TRF2 in complex with ds-DNA. **(E)** Double Myb domain from budding yeast Rap1 in complex with ds-DNA. **(F)** Alignment of TRF1 and TRF2 telobox sequences across different species allows definition of a consensus sequence and thus of a different signature between them (for details on the species used for the alignment, see Poulet et al., 2012).

For ss-DNA binding, the main structural theme is the OB-fold (oligonucleotide/oligosaccharide binding fold) (Figure 4A). So far, all the telomeric complexes identified relied on this domain for ss-DNA binding. Furthermore, proteins or protein complexes that carry this motif are essential for telomere protection (for review, see Lewis and Wuttke, 2012). OB-folds are characterized by a combination of low sequence conservation with high three-dimensional structure conservation (Flynn and Zou, 2010). They form a beta barrel of ∼100 residues composed of two three-stranded antiparallel β-sheets, the first β-strand belonging to both β-sheets, they are capped by an α-helix at one end, and present a binding cleft at the other end. The connecting loops between β-sheets strongly vary between species in terms of sequence, length, and conformation, contributing to the binding specificities of the OB-folds (Murzin, 1993) (Figure 4B).

In TEBP three OB-folds are involved in DNA interaction, two from TEBP-β, and the third one from TEBPα (Horvath et al., 1998). Orthologs of the TEBP heterodimer have been described in fission yeast (Pot1-Tpz1, Miyoshi et al., 2008), mammals (POT1-TPP1, Wang et al., 2007), budding yeast (Cdc13-Stn1-Ten1, Bertuch and Lundblad, 2006), and plants (CTC1-Stn1, Surovtseva et al., 2009).

Known telomeric ds-DNA binding proteins mainly rely on a Myb/Sant domain to interact with DNA (Figure 4C). Alignments of Myb/Sant domains across species reveal that the telomeric motif can be distinguished from the ones held by non-telomeric proteins, hence the term of telobox to design these domains (Bilaud et al., 1996, 1997; Giraud-Panis et al., 2010a). Teloboxes are usually composed of three helices, the third helix displaying conserved residues that allow specific recognition of telomeric DNA. Three-dimensional structures of teloboxes are available from human TRF1 and TRF2 (Fairall et al., 2001; Court et al., 2005; Hanaoka et al., 2005) (Figure 4D) and from plants (Sue et al., 2006; Ko et al., 2008, 2009). The three helices that characterize the Myb domain adopt similar conformation and the overall scheme of protein-DNA interaction is preserved, although some minor differences in the sequences can be used to distinguish between TRF1 and TRF2 teloboxes (Poulet et al., 2012) (Figure 4F). In plants, several proteins able to bind telomeric DNA *in vitro* have been identified (for review, see Watson and Riha, 2010) but the role, if any, of these proteins on telomeres is still largely unknown. The domains organization of NgTRF1 (Tobacco), RTBP1 (Rice), and AtTRP1 (*A. thaliana*) is similar to the one found in the vertebrates telobox containing proteins. However, a fourth helix is present in the Myb domain that was described to be essential for DNA interaction, and the loop between helix three and four carries an additional Arginine that could explain specificity of plant TRFs to the TTTAGGG sequence (Sue et al., 2006; Ko et al., 2008, 2009). In addition to the TRF-like family, plants encode SMH-like (single myb histone) proteins that bind plant telomeric ds-DNA through a N-terminal Myb domain (Marian et al., 2003; Schrumpfova et al., 2004) and possess a central H1/5 domain involved in the formation of dimers or multimers (Karamysheva et al., 2004). Most of telobox proteins, including Tbf1 from *S. cerevisiae* that binds the TTAGGG repeats found immediately adjacent to the terminal TG1-3 repeats in this organism (Fourel et al., 1999), contain a conserved dimerization domain located close to the N-terminal. In plant TRFs, homodimerization relies of the additional C-terminal region found at the end of the Myb domain (Karamysheva et al., 2004; Watson and Riha, 2010). It appears therefore that all telomere proteins containing a single Myb domain have developed structural strategies that lead to dimerization. Although dimerization has been proved to improve efficiency of binding (Fairall et al., 2001), and selectivity for long tract of telomeric sequence (Karamysheva et al., 2004), the precise role of this dimerization remains poorly documented. In addition to the minimal telobox domain, the N-terminal basic domain of TRF2 also interacts with four-stranded DNA junctions, Holliday junctions (Poulet et al., 2009).

The DNA binding domain of the budding yeast Rap1 protein contains two Myb domains unrelated to teloboxes (Figure 4E). The first Rap1 Myb domain corresponds to a canonical Myb domain, and the second Myb domain has an additional fourth helix. In addition, a 30 residues loop located at the C-terminus of the second Myb is responsible for Rap1 wrapping around the DNA molecule (Konig et al., 1996; Matot et al., 2012); a wrapping that was shown recently to be involved in the integrity of the complex (Matot et al., 2012).

In *Drosophila*, where a functional telomere can be established in the absence of a specific DNA sequence, the DNA extremities are also protected by a specific telomeric complexes named Terminin including the proteins HOAP (HP1/ORC Associated Protein), Moi (Modigliani), Ver (Verrocchio), and HipHop (reviewed in Raffa et al., 2011). How Terminin is specifically recruited at the ends of *Drosophila* chromosomes is still elusive. The protein HOAP was shown to bind directly to ds-DNA, probably through an HMG-like domain (Shareef et al., 2001). Interestingly, the ss-DNA binding protein Ver that binds the telomeric 3′ overhang contains an OB-fold that is related to the one of human STN1 (Raffa et al., 2011). The conservation of this motif across species is intimately linked to the versatility of the OB-fold which structure can be adapted to the variability of the DNA or proteins targets.

## Nucleosomes: A Role at the End?

In most organisms telomeric DNA is organized in a chromatinized structure but exceptions exist in lower eukaryotes where there is a lack of nucleosomal organization (Gottschling and Cech, 1984; Wright et al., 1992). In higher eukaryotes, telomeric DNA is organized in an unusual chromatin structure characterized by tightly packed nucleosomes, in which the nucleosomal repeat length (NRL) is always ∼40 bp shorter than the one of the bulk chromatin (Lejnine et al., 1995).

Although the organization of telomeric nucleosomal arrays *in vivo* could be modulated by other constraints, like specific protein-DNA interactions, the specific sequence-dependent features of telomeric DNA seem to represent a crucial determinant for chromatin organization both in terms of nucleosomal positioning and spacing. Indeed, telomeric nucleosome exhibit a significantly higher mobility compared to nucleosomes organized on average sequences (Pisano et al., 2007) and shorter NRL can be observed in reconstituted chromatin fibers using telomeric DNA (Pisano et al., 2006). It is also worth noting that vertebrate telomeric DNA has the lowest affinity for nucleosome formation among numerous DNA sequences studied (Cacchione et al., 1997; Rossetti et al., 1998; Filesi et al., 2000). This low affinity can be attributed both to the global straightness of telomeric DNAs and their flexibility. In addition, positioning of histones on DNA is optimal when the preferred sequence is regularly spaced with a helical periodicity of 10.2 bp (rotational positioning). In most cases, the periodicity of telomeric DNA is dramatically out of phase, thus causing a significant increase in the free energy of nucleosome formation. Moreover, since the recurrence of the repeats giving rise to isoenergetic and, as a consequence, equiprobable multiple positioning sites, nucleosomes have also no preferred position (no translational positioning) (Rossetti et al., 1998; Pisano et al., 2006).

Higher-order organization, i.e., the chromatin fiber, has also been studied. Nikitina and Woodcock (2004), using chromatin from chicken erythrocytes and mouse lymphocytes, visualized putative chromatin t-loops which diameter (roughly 30 nm) had no apparent difference compared to linear fibers in bulk DNA. However, the fine structure of the fiber is expected to be somewhat different because it depends on the length of the linker DNA and on its orientation in space (Routh et al., 2008) – an organization that involves the linker histone H1.

Low level of linker histones is generally associated with shorter NRL (Woodcock et al., 2006). Based on this correlation, the content of H1 on telomeric chromatin is expected to be lower than on bulk chromatin. Analyzing the telomeric chromatin of *A. thaliana* (Ascenzi and Gantt, 1999), H1 was indeed found associated to telomeric nucleosomes with a decreased stoichiometry with respect to bulk chromatin. Similarly, a low ratio H1/nucleosome was observed in telomeres from human adult fibroblasts (Parseghian et al., 2001) and in HeLa cells (Dejardin and Kingston, 2009). On the other hand, the analysis of telomeric chromatin from rat hepatocytes revealed a short telomeric NRL, but the same amount of H1 histone in telomeric and bulk chromatin (Bedoyan et al., 1996). Thus the relative amounts of H1 seem to vary depending on the species. It remains, that histone H1 is a component of telomeric chromatin and likely plays an important role in the structure of the telomeric chromatin fiber.

Histone post-translational marks of mouse telomeres resemble those of constitutive heterochromatin (reviewed in Schoeftner and Blasco, 2009). However, this is not the case in all organisms. Studies in *A. thaliana* by ChIP-seq, recently revealed histone marks that more closely resemble those found on repressed or lowly expressed euchromatic genes (Vaquero-Sedas et al., 2011, 2012). With the same technique, the most significant modifications found in the telomeres of human CD4^+^ T cells were H2BK36me1 and H3K4me3 (more euchromatic) while H3K9me3 and H4K36me3 marks (more heterochromatic) were less represented (Rosenfeld et al., 2009). Studies of histone marks at telomeres of polytene chromosomes in *Drosophila* also reveal a mix of chromatin signatures (H3K9me3, H3K4me3) (Andreyeva et al., 2005). Hence, a heterochromatin profile of histone marks does not seem to be a hallmark of telomeric chromatin.

Telomeres have also been shown to contain variants of H3 and H2A. H3.3 is a highly conserved histone variant among eukaryotes (Malik and Henikoff, 2003), diverging from the canonical H3 by just four residues. Its role has been generally associated with active chromatin (Ahmad and Henikoff, 2002; McKittrick et al., 2004). In mouse ES cells telomeres, it was recently reported to be involved in the plasticity of telomeric chromatin (Wong et al., 2009; Goldberg et al., 2010), and in TERRA transcription inhibition (Goldberg et al., 2010). H3.3 recruitment on telomeres is specifically mediated by ATRX, a SNF2-like ATP-dependent chromatin factor, coupled with Daxx (Wong et al., 2009; Goldberg et al., 2010; Lewis et al., 2010). A very recent study on human embryonic kidney cells revealed that ATRX also regulates, negatively in that case, the telomeric recruitment of macroH2A.1, a vertebrate histone variant of H2A (Ratnakumar et al., 2012). The authors hypothesized that this regulation of macroH2A1 at telomeres might have an impact on telomere integrity. Another H2A variant, H2A.Z, has been detected on *Drosophila* telomeres where it was shown to rescue some defects in telomere capping (Rong, 2008). Another peculiar case of telomeric histone variant is H3V in *Trypanosoma brucei*, a variant that shares ∼50% identity with H3, which is enriched at telomeres, although the role of this enrichment is not yet established (Lowell and Cross, 2004).

The human Shelterin protein TRF1 was shown to form stable ternary complexes *in vitro* with the telomeric nucleosome. This binding caused structural alterations in the nucleosomes (Galati et al., 2006) leading to an increased mobility (Pisano et al., 2010). Additionally, the ability of hTRF2 to influence the telomeric chromatin structure was tested in cells with contrasted results. On one hand, Benetti and colleagues reported a decrease in the amount of H3 and H4 histones in primary murine keratinocytes overexpressing TRF2. This was correlated to a decrease in nucleosomal spacing compared to wild type cells (Benetti et al., 2008). The same alteration of telomeric nucleosomal organization was later found in different human cancer cell lines overexpressing TRF2 (Galati et al., 2012). On the other hand, Wu and de Lange (2008) observed no differences between TRF2-deficient mouse embryonic fibroblasts and wild type cells. A possible explanation for this divergence might be found in the use of different cell lines, in which the impact of TRF2 expression on telomeric organization and function could be different. Alternatively, telomeric chromatin may respond differently to overexpression (Benetti et al., 2008; Galati et al., 2012) versus depletion (Wu and de Lange, 2008) of TRF2.

Nonetheless, nucleosomes, TERRA and capping proteins must coexist in some way along telomeres. It is yet unclear if nucleosomal and nucleosome-free/capping protein-bound domains are interspersed. A widespread view is that nucleosomes occupy the centromere-proximal part of telomeres, the capping protein being concentrated at their very end (Cohen and Blackburn, 1998; Freitas-Junior et al., 1999; Figueiredo et al., 2000).

In summary, the fact that nucleosomes are absent at short telomeres of some unicellular organisms suggests that they may be detrimental for telomere functions. In organisms where nucleosomes are naturally present at telomeres, how they cohabit with capping proteins and whether they play a role in telomere function are still unanswered and fascinating questions.

## Telomeric Heterochromatin: A Backup Protection?

Direct link between capping proteins and heterochromatin factors seems a widespread phenomenon. In *S. cerevisiae*, the archetypal example, Sir proteins interact with Rap1 in order to initiate the spreading of silent chromatin into the subtelomeric region (Ottaviani et al., 2008; Li, 2010). However, despite a slight shortening of telomeres, the disruption of Sir genes does not affect telomere protection in *S. cerevisiae* (Palladino et al., 1993), showing that the heterochromatin initiated at telomeres is not strictly required for telomere capping, at least in budding yeast.

Nevertheless, in *Drosophila* telomeres, end protection depends upon the major heterochromatin factor HP1 (Fanti et al., 1998), which interacts with the HOAP, HipHop, and Moi proteins from the Terminin complex (reviewed in Raffa et al., 2011). In accordance with an enrichment of tri-methylated H3K9 at mouse telomeres, a terminal binding of HP1 was observed (Garcia-Cao et al., 2004). In human cells, HP1α can also be found at telomeres (Koering et al., 2002) whilst HP1γ has been shown to interact with TIN2 to promote cohesion during S phase and elongation by the telomerase enzyme (Canudas et al., 2011; Houghtaling et al., 2012).

A compelling evidence for a role of heterochromatin in telomere protection stems from studies in *S. pombe* showing that a massive rearrangement of heterochromatin blocks to chromosome ends can rescue the loss of telomeric repeats (Jain et al., 2010). This phenomenon defines a mode of telomerase-independent telomere maintenance mechanism dubbed HAATI (for Heterochromatin Amplification-mediated And Telomerase-Independent). Strikingly, HAATI is independent of the telomeric ds-DNA capping protein Taz1 but requires Ccq1 and the ss-DNA binding factor Pot1. This together with fact that Ccq1 interacts with the SHREC silencing complex (Snf2/Hdac-containing repressor complex) (Sugiyama et al., 2007), suggests that Ccq1 is recruited by SHREC in HAATI cells, providing a terminal anchor for Pot1. This would provide a backup mechanism for the recruitment of capping proteins at telomere and for end protection in the absence of telomeric DNA. Whether a similar epigenetic mechanism acts at *Drosophila* telomeres or at other heterochromatic telomeres will certainly be an important issue to address.

## Nuclear Localization and Dynamics of Telomeres: Keep Attached!

**Figure 5:**
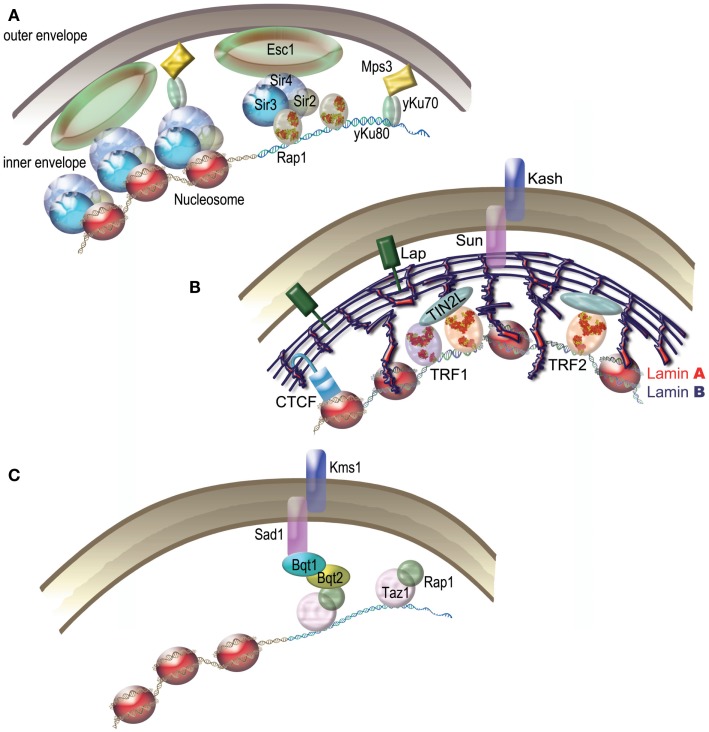
**Telomeric nuclear tethering**. **(A)** *S. cerevisiae*. Schematic representation of Sir4–Esc1 anchoring and yKu-Mps3 anchoring pathways of yeast telomeres. **(B)** Humans. Several pathways are proposed to tether human telomeres to the nuclear matrix. The tethering of human telomeres was proposed to depend on TIN2L. Lamina, a component of the nuclear matrix, is linked to the nuclear membrane by LAP proteins and by a Sun/Kash type complex. CTCF participates in the nuclear localization of subtelomers via lamina. **(C)** *S. pombe* Bouquet. During meiosis, Bqt1 and Bqt2 proteins join Rap1 and Taz1 to Sad1, thus tethering telomeres to the nuclear envelope. The Sad1-Kms1 complex anchors the whole structure to microtubules.

In the late nineteenth century, Carl Rabl reported that telomeres of interphase nuclei in salamander cells localize close to the nuclear envelope (NE) on one side of the nucleus, while centromeres occupy the other side. This organization, since then called the “Rabl” organization, has been observed in various species from yeast, to plants and animals (Cowan et al., 2001; Nagai et al., 2010). In plants, the size of the genome seems to condition the presence of the Rabl arrangement (Cowan et al., 2001), while it is particularly striking in *D. melanogaster*. The budding yeast *S. cerevisiae* displays a Rabl-like organization where telomeres are clustered and tethered at the NE in three to eight foci (Taddei et al., 2010) (Figure 5A). Tethering and clustering are functionally distinct phenomenon (Mondoux et al., 2007; Horigome et al., 2011). Tethering was suggested to protect against illicit recombination probably during S phase (Schober et al., 2009) while clustering seems to be more important for silencing thanks to the localized concentration of Sir proteins it generates (Maillet et al., 1996). Association of telomeres with the NE is also a feature observed in *S. pombe* but, here, the telomeres are dispersed along the envelope (Chikashige et al., 2010).

Nonetheless, association of telomeres to the NE during interphase is far from being universal. Even in plants where the Rabl organization is common, exceptions exist such as *A. thaliana* where telomeres are associated with the nucleolus throughout interphase (Roberts et al., 2009). A lack of consensus is also observed in mammals where telomeres subnuclear localization depends on species, cell type, oncogenic status, and even identity of the telomere (Weierich et al., 2003; Chuang et al., 2004; Louis et al., 2005; Ottaviani et al., 2009; Arnoult et al., 2010). A tendency for the inner space of the nucleus has been sometimes observed (Arnoult et al., 2010) and some subtelomeric sequences have been shown to affect telomere positioning (Ottaviani et al., 2009) (Figure 5B). Perinucleolar localization has also been reported for acrocentrics (Ramirez and Surralles, 2008). Whatever their precise subnuclear localization, telomeres do not appear to be free to roam throughout the nucleus. Indeed, human telomeres are associated to the nuclear matrix – an insoluble fraction of the nucleus (de Lange, 1992; Luderus et al., 1996). This association was recently proposed to involve a newly identified isoform of TIN2 (TIN2L) (Kaminker et al., 2009). Another candidate for mediating telomere subnuclear attachment is A-type lamin. Indeed, reducing the level of A-type lamins in human epithelial cervix carcinoma C33-A cells suppresses the ability of a single D4Z4 repeat to cause peripheral positioning of an associated telomeres (Ottaviani et al., 2009). Telomeres length and positioning is altered in mouse cells depleted in A-type lamins (although more peripheral in that case). In addition, telomere shortening has been observed in the case of several Lamin A mutations such as in the progeroid Hutchinson Gilford syndrome, still strengthening the link between A-type lamins and telomeric functions (for review, see Gonzalez-Suarez and Gonzalo, 2010).

Probably as a consequence of the attachment of telomere to a fixed subnuclear structure, most of the telomeres of human and mouse cells exhibit constrained diffusive movements (Molenaar et al., 2003; Dimitrova et al., 2008; Wang et al., 2008; De Vos et al., 2009; Jegou et al., 2009; Nagai et al., 2010). Telomeric movements increase during recombination in ALT cells (Molenaar et al., 2003; Jegou et al., 2009), transcription (Arora et al., 2012) and in case of deprotection (Dimitrova et al., 2008; Wang et al., 2008). Similarly, telomeres in the budding yeast relocalize to nuclear pores to be elongated and/or repaired (Therizols et al., 2006; Abdallah et al., 2009; Khadaroo et al., 2009; Oza et al., 2009; Ferreira et al., 2011; Nagai et al., 2011).

A spectacular example of telomere NE association occurs during meiotic prophase I, where telomeres of most species cluster to form a specialized structure called Bouquet (notable exceptions are *C. elegans* and *D. melanogaster* which use alternative methods for chromosome pairing; Tsai and McKee, 2011). Most of our knowledge on the molecular mechanisms of Bouquet comes from studies in the fission yeast (Figure 5C). Successive interactions between Rap1, Taz1, Bqt1/Bqt2, Sad1, and Kms1 link telomeres inside the nucleus to microtubules in the cytoplasm, thus allowing movements that are powered by a meiosis-specific dynein motor (Chikashige et al., 2007). Sad1 and Kms1 belong to the evolutionary conserved Linker of Nucleoskeleton and Cytoskeleton (LINC) complex. In many species this complex is based on the interactions between SUN (*S*ad1, *Un*c-84 from *C. elegans*) domain containing proteins and KASH (Klarsicht from *Drosophila*, ANC-1 from *C. elegans*, Syne Homology from mammals) domain containing proteins. When these domains are not present functional equivalents can be found. There are five SUN-domain proteins in humans (SUN1 and SUN2 are the inner membrane components) and three KASH domain proteins (Nesprin/SUNE-1, -2, and -3) and in mice SUN1 and SUN2 have been shown to be involved in tethering telomeres to the NE (Zhou et al., 2012). Strict homologs of the fission yeast Bqt1 and Bqt2 have not been identified in other species yet, although the budding yeast protein Ndj1 has been proposed to be a functional equivalent. Indeed, similarly to the Bqt1/Bqt2 proteins, Ndj1 is required for bouquet formation and telomere mobility and was found to bind Mps3 (a SUN-domain protein) by double-hybrid assay (Hiraoka and Dernburg, 2009 and references within).

On the telomeric side, recent data on mice show that contrary to *S. pombe* Rap1, mouse RAP1 is not involved in telomere attachment or clustering during meiosis (Scherthan et al., 2011). TRF2 has been observed on telomeres during the meiotic Prophase I and II in mouse spermatocytes (Scherthan et al., 2000; Siderakis and Tarsounas, 2007) but its function in Bouquet formation, if any, is unknown. The role of the Bouquet itself is also rather elusive. Promoting homologous chromosome pairing has been proposed but recent data in budding yeast suggest that chromosome movements rather than telomere clustering may be more important (Lee et al., 2012). In accordance for other roles, telomere clustering in the Bouquet was shown to be crucial for the maturation of the spindle pole body in *S. pombe* (Tomita and Cooper, 2007).

## Topological Stress: An Emerging Theme in Telomere Biology

The formation at telomeres of t-loops, Holliday junctions, and G4 as well as the tight attachment to subnuclear structures are expected to block the rotation of telomeric DNA. Hence, transcription and replication may cause important topological problems at chromosome ends. Several recent works support this view and suggest that the resolution of topological problems is at the heart of telomere biology.

**Figure 6:**
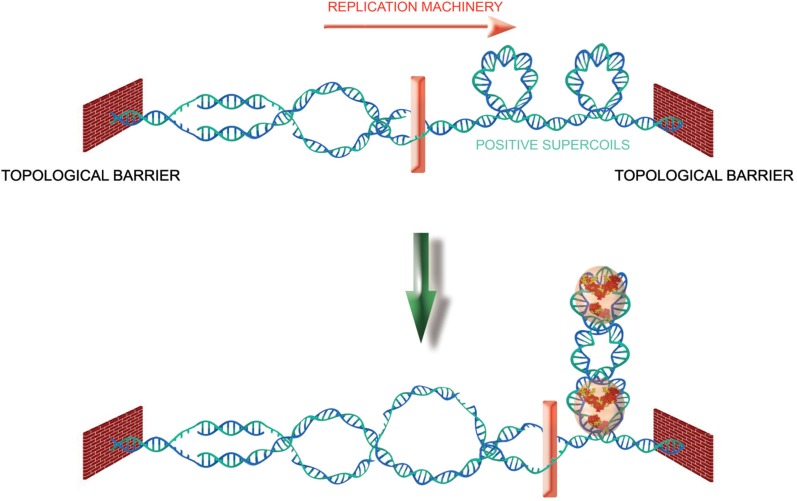
**TRF2 as a topological stress sensor**. Due to topological constraints, during replication, opening of the double helix produces pre-catenanes behind the fork and positive supercoils ahead. TRF2 binding to these positive supercoils would allow the recruitment of enzymatic activities (Apollo for instance) that would help to relieve the topological stress.

Topoisomerase I is a constitutive member of the telomeric complex of the linear chromosomes and plasmids in the *Streptomyces* bacterial species (Bao and Cohen, 2004). It is thought to resolve the topological constraints that arise from the association between different telomeres though interactions of covalently bound telomeric complexes (Tsai et al., 2011). Positive supercoiling at telomeres of another bacterial species, *Borrelia*, is a driving force that allows resolution of dimer telomeric junctions formed during replication (Bankhead et al., 2006; Chaconas and Kobryn, 2010). In unicellular eukaryotes, topoisomerase 2 was shown to play a role in telomeres segregation in the fission yeast (Germe et al., 2009). In human cells, the telomeric G4-targeting molecule RHPS4 potentiates the anti-tumor efficacy of TOPO I (topoisomerase I) inhibitors in preclinical models (Leonetti et al., 2008; Biroccio et al., 2011) and TRF2 protects against the damages caused by topoisomerase 2 poisons (Klapper et al., 2003; Zhang et al., 2008). Furthermore, topoisomerase 2α is required for telomere protection in a pathway involving TRF2 and its partner Apollo (Ye et al., 2010). Interestingly, TRF2 decreases the amount of topoisomerase 2α needed for a proper end protection, suggesting a model in which TRF2 relieves the excess of topological stress generated during telomere replication. Since TRF2 is able to wrap DNA in a right-handed manner (Amiard et al., 2007) and preferentially binds to positively supercoiled DNA substrates, it might serve as a topological stress sensor, warranting rapid access to and coordinating the action of multiple enzymatic activities to prevent aberrant topological resolution (Figure 6). This function of TRF2 might be tightly regulated during cell cycle. For instance, the wrapping ability of TRF2 is abrogated by its binding to TERRA (Poulet et al., 2012).

## Conclusion: Capping Proteins and Topological Stress as Universal Features of Telomere Identity?

**Figure 7:**
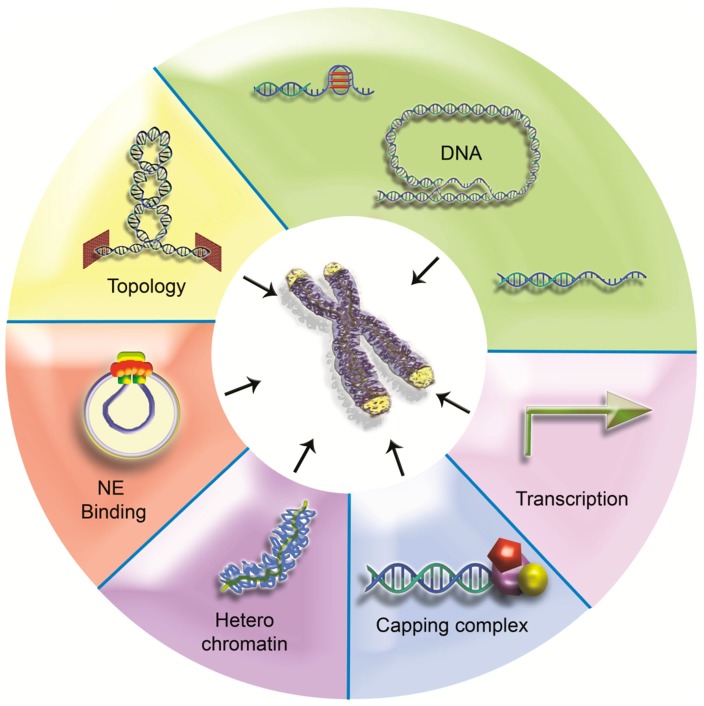
**One terminal problem, different solutions?** Some telomeric features are universal but others are less conserved. Telomeric DNA can adopt diverse structures (5′ overhang, G4, and t-loop for examples) and can even lack repeats (*Drosophila*, HAATI telomeres). Failing specific sequences, heterochromatin can provide a backup system for protection. Although heterochromatic properties have been linked with peripheral localization in budding yeast, this telomere positioning is not a widespread feature. Conversely, transcription seems to be shared by all telomeres studied so far. Capping proteins are also major components of telomeres, particularly overhang binding proteins. Recent data suggest that topological issues may be of particular relevance at telomeres. Topological stress may constitute a conserved signaling pathway to recruit end capping proteins.

Through the years much has been learned about how telomeres provide solutions to the problems arising at chromosome ends. Considering the importance of these chromosome elements, it is amazing how diverse these solutions are. Comparing the various levels of telomere organization, one can observe that some known telomeric characteristics are widespread among species while some others are not (Figure 7).

The presence of a specific telomeric DNA sequence does not seem to be a universal requirement for telomere function, suggesting that epigenetic determinants can operate to protect chromosome ends. This is the case in *Drosophila* but also in HAATI *S. pombe* cells. The presence of arrays of short DNA repeats, as present in many organisms, is linked to the maintenance of telomeres by a telomerase-based mechanism and may have been conserved through evolution due to the sequence specificity of capping proteins. Nucleosomes do not seem also to be a universal piece of the telomere puzzle since they are excluded from telomeric DNA in several species. Nevertheless, in some organisms, higher-order organization of chromatin, like heterochromatin, can contribute to chromosome end protection, i.e., by recruiting capping proteins as proposed for HAATI cells.

One feature that appears to be universally conserved and absolutely required for chromosome end capping is the recruitment at the very end of the chromosomal DNA of non-histone protein complexes. These complexes are quite diverse in form and composition but always appear to contain protein(s) bound to the 3′ overhang. These proteins are clearly central for telomere biology.

We would like here to hypothesize that the inability to rotate the DNA end is also a universal feature of telomeres. It is quite amazing to observe that in all species where it has been studied, telomeric DNA appears constrained. In bacteria with linear chromosomes, this is achieved through the covalent binding of terminal proteins or a covalent link between the 3′ and the 5′ ends. In eukaryotes, this is likely to be the consequence of the various higher-order structures that can be adopted by telomeric chromatin (G4, t-loop, subnuclear attachment sites…). It is thus tempting to propose that a high level of topological constraints during telomere replication and transcription constitutes an ancestral signal for the recruitment of capping proteins, allowing the coupling between regulation of topology and protection of chromosome ends.

## Conflict of Interest Statement

The authors declare that the research was conducted in the absence of any commercial or financial relationships that could be construed as a potential conflict of interest.
